# Open vs laparoscopic repair of abdominal hernia: a case control study in over 60 years old patients

**DOI:** 10.1186/1471-2482-13-S1-A19

**Published:** 2013-09-16

**Authors:** Massimiliano Fabozzi, Rosaldo Allieta, Luciano Grimaldi, Stefano Reggio, Bruno Amato, Michele Danzi

**Affiliations:** 1 Department of General Surgery . “U. Parini” Hospital, Aosta, Italy; 2 Department of Specialized Surgery, Division of Gastrointestinal Surgery Rehabilitation of Election and Emergency. “Federico II” University, Naples, Italy

## Background

**Figure 1:**
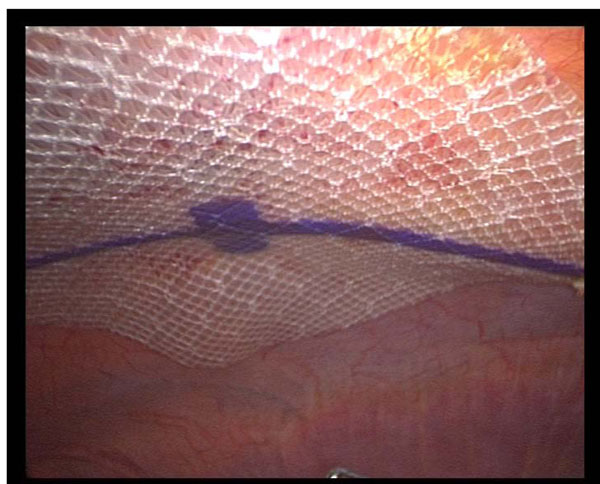
**intraoperative image** Laparoscopic repair of incisional hernia.

About 15 % of patients who have been undergone to a laparotomy may develope abdominal wall hernia and the risk increases with age. In last years the Laparoscopic treatment of ventral hernia (LVHR) is becoming increasingly widespread in surgical community thanks to the good outcomes of this technique [1,2]. The aim of this study was to describe the experience of our surgical centers in order to establish the safety, efficacy, and feasibility of LVHR using composite mesh and tacks (Figure 1) compared to the open technique (OVHR).

## Methods

Between January 2001 and March 2013, 523 patients were admitted to the Specialistic Surgery Centers ( Aosta " U. Parini Hospital and Naples "Federico II " Hospital ) and treated for abdominal wall hernia. Two groups (Open and Laparoscopic Repair), each one of 100 patients, with similar characteristics have been selected from these(mean age, sex, BMI, ASA risk and for type and size of surgical defects, Table 1): the first group was treated by laparoscopic repair and the second by open hernia repair. Mean age was 66 years old (range: 60-72) in open group and 68 years old (range: 63-73) in Laparoscopic group. Study outcomes were : operative time, complications, postoperative pain, analgesic therapy duration, intestinal function restoration, mean hospital stay, mortality and rate of recurrence at oneyear follow-up.

**Table 1:** Patients data and operative parameters

	Open abdominal wall hernia repair	Laparoscopic abdominal wall hernia repair
**Patients number**	100	100
**Age (years)**	65.4±4.5	67.7±3.1
**Sex**	56 F / 44 M	49F / 51M
**BMI (kg/m^2^)**	27.1±1.9	29.3±3.6
**Asa i**	43	32
**Asa ii**	47	49
**Asa iii**	10	19
**Wall defects size (cm)**	12.6±9.2	11.4±9.7
**Ng tube removal**	after the operation	after the operation
**Urinary catheter removal**	evening of surgery	after the operation (only in parapubic repair)
**Water assumption**	evening of surgery	evening of surgery
**Time of refeeding**	3rd p.o. day	1st p.o. day

## Results

Outcomes data are shown in Table 2 . In LVHR group the 55% of patients presented incisional hernia and 45% epigastric or umbilical hernias. In OVHR group the 52% of patients presented incisional hernia and 48% epigastric or umbilical hernias. The mean size of surgical defects was 11.4+9.7 cm in Laparoscopic group and 12.6+9.2 cm in Open group. Mean operative time was 61+22 min in Laparoscopic group and 105+27 min. The post-operative complications rate was 14% in Open group and 5% in Laparoscopic group. Patients who underwent LVHR presented a more rapid restoration of intestinal function, less postoperative pain and subsequently shorter analgesic therapy compared with the OVHR group. Postoperative complication rate is higher in the OVHR than LVHR group but the mortality rate was 0% for both tecniques. Mean hospital stay expressed in days is significantly reduced in LVHR. At one-year follow-up, we observed 7% in OVHR vs 4% in LVHR of hernia recurrence.

**Table 2:** Outcomes

Outcomes	Open abdominal wall hernia repair	Laparoscopic abdominal wall hernia repair
**Operative time** (min.)	105±27	61±22
**Intra-operative complications**	0	0
**Laparotomy size** (cm)	16±7	No
**First peristalsis** (days)	2.1±0.9	1.1±0.7
**First defecation** (days)	3.1±1.6	1.6±1.3
**Permanence of drain** (days)	2.3±1.6	No
**Post-operative pain** (VAS pain scale)	6.6	1.7
**Anesthetic tap block**	No	Yes
**Analgesic duration terapy**(days)	4. 8±1.5	1.1±1.5
**Post-operative complications** (number, rate)	14 (14%)	5 (5%)
**Hospital stay** (days)	5.6±1.2	1.9±1.8
**Mortality**	0	0

## Conclusions

LVHR is an effective and safe procedure with very low morbidity and recurrence rates [3,4]. It is associated with less postoperative pain and respiratory complications in over 60 years old patients thanks to less p.o. pain that doesn't compromise the diaphragmatic respiratory movements [5].

By our experience and the datas of Literature we can conclude that the Laparoscopic treatment of abdominal wall hernias (Incisional and not) presents more advatages compared to Open procedures related to reduced global complications and hospital stay with better comfort of patients.

**Table 3:** Complications

	Open abdominal wall hernia repair	Laparoscopic abdominal wall hernia repair
**Hematoma**	6 %	1 %
**Seroma**	4 %	3 %
**Visceral lesions**	0	0
**Sub-occlusion**	0	0
**Respiratory infections**	1 %	0
**Infections of prosthesis**	0	0
**Fascial necrosis**	0	0
**Postop. Pain (6 mesi)**	3 %	1 %
**Recurrence**	7 %	4 %
**Mortality**	0	0

## References

[B1] Itani KMF Hur K Kim L Anthony T Neumayer L Comparison of laparoscopic and open repair with mesh for the treatment of ventral incisional hernia Arch Surg 2010 145 4 322 8 10.1001/archsurg.2010.18 20404280

[B2] Eker HH Hansson BM Buunen M Janssen IM Pierik RE Hop WC Bonjer HJ Jeekel J Lange JF Laparoscopic vs. open incisional hernia repair: a randomized clinical trial JAMA Surg 2013 148 3 259 63 10.1001/jamasurg.2013.1466 23552714

[B3] Amato B Moja L Panico S Shouldice technique versus other open techniques for inguinal hernia repair (Review) Cochrane database of systematic reviews (online) 2012 4 CD001543 10.1002/14651858.CD001543.pub4 PMC6465190 22513902

[B4] Cuccurullo D Piccoli M Agresta F Magnone S Corcione F Stancanelli V Melotti G Laparoscopic ventral incisional hernia repair: evidence-based guidelines of the first Italian Consensus Conference Hernia 2013 10.1007/s10029-013-1055-1 23400528

[B5] Rea R Falco P Izzo D Leongito M Amato B Laparoscopic ventral hernia repair with primary transparietal closure of the hernia defect BMC Surgery 2012 12 Suppl.1 S5 23173597 10.1186/1471-2482-12-S1-S33 PMC3499268

